# Association of Psilocybin Use in Adolescents with Major Depressive Episode

**DOI:** 10.1192/j.eurpsy.2022.837

**Published:** 2022-09-01

**Authors:** K. Shah, C. Trivedi, D. Kamrai, M. Akbar, W. Tankersley

**Affiliations:** 1 Griffin Memorial Hospital, Psychiatry, Norman, United States of America; 2 Texas Tech University Health Science Center at Permian Basin, Psychiatry, Midland, United States of America

**Keywords:** MDD, Psilocybin, Depression, Adolescents

## Abstract

**Introduction:**

Psilocybin is a psychedelic drug found in mushrooms, often referred to as magic mushrooms due to its visual and auditory hallucinations effects upon ingestion. It is a Schedule I drug per DEA, and the FDA has not approved psilocybin for medicinal purposes. However, recent studies have shown promising therapeutic use to treat depression.

**Objectives:**

To identify current use, prevalence, and its association with depression in adolescents.

**Methods:**

The National Survey on Drug Use and Health survey data from 2008-18 studied adolescent data (12-17 years), who responded, “ever used psilocybin (mushrooms)” and “lifetime major depressive episode (MDE).” The association between the psilocybin use and MDE status was analyzed in SAS 9.4 through multivariate logistic regression for odds ratio (OR) and 95% confidence interval (CI).

**Results:**

A total of 172745 adolescents were included in this study, of which 2469 ever used psilocybin in their lifetime, and 170276 responded no lifetime use. The psilocybin ever lifetime users were 17 years old (42%vs.17%,p<0.001), male (60%vs.51%,p<0.001), and non-Hispanic White (71%vs.55%,p<0.001) in comparison to non-users. Among psilocybin user group, 31% of respondents had lifetime MDE, compared to 16% of the lifetime psilocybin non-user group participants (p<0.001). The odds of association of psilocybin use among participants with MDE were 2.17 times compared to those without MDE (CI: 1.93-2.44,p<0.001).

**Conclusions:**

We identified a significant association between psilocybin use and MDE among adolescents, which raises public health concerns about its illegal use, abuse, and toxicity potential. Future clinical studies should assess its clinical safety, efficacy, and addictive properties.

**Disclosure:**

No significant relationships.

